# Effects of Low-Dose Testosterone Undecanoate Treatment on Bone Mineral Density and Bone Turnover Markers in Elderly Male Osteoporosis with Low Serum Testosterone

**DOI:** 10.1155/2013/570413

**Published:** 2013-03-04

**Authors:** Yan-Jiao Wang, Jun-Kun Zhan, Wu Huang, Yi Wang, Yuan Liu, Sha Wang, Pan Tan, Zhi-Yong Tang, You-Shuo Liu

**Affiliations:** Geriatric Department of the Second Xiang-Ya Hospital, Institute of Aging and Geriatric Research, Central South University, No. 139 Middle Renmin Road, Changsha, Hunan 410011, China

## Abstract

This prospective 2-year, single-center, randomized, placebo-controlled, open-label clinical trial was performed to evaluate the efficacy of low-dose testosterone undecanoate (TU) treatment on bone mineral density (BMD) and biochemical markers of bone turnover in elderly male osteoporosis with low serum testosterone. A total of 186 elderly male osteoporosis patients with low serum testosterone were randomized into three groups: low-dose TU (20 mg, per day), standard-dose TU (40 mg, per day), and placebo group with a 24-month followup. Since the 6th month in standard-dose TU group or since the 12th month followup in low-dose TU group and throughout the study, lumbar spine and femoral neck BMD and serum levels of free testosterone, estradiol, and bone alkaline phosphatase significantly increased. There were no significant differences between groups of low-dose TU and standard dose TU in the percentage of changes of these data since the 18th month followup and throughout the study. No side effects on prostate glands including prostate specific antigen were found. In conclusion, low-dose TU (20 mg, per day) may be a cost effective and safe protocol for treating elderly male osteoporosis with low serum testosterone.

## 1. Introduction

With the aging of the population, osteoporosis in men is becoming an increasingly important public health problem. Aging men lose bone mineral density (BMD) at a rate of approximately 1% per year [1]. Johnell and Kanis [2] recently updated the worldwide prevalence of osteoporotic fractures using data from published sources. One in five men over the age of 50 will suffer an osteoporotic fracture during their lifetime. Of the annual incidence of 9 million fractures, 39% were in men. Thirty percent of hip fractures occurred in men. Hip fractures are the most main reason leading to death or disability in all kinds of fractures. 

As is the case in women, the mainstays of therapy for osteoporosis in men are the bisphosphonates. For patients at high risk of fracture, use of teriparatide in men has also been approved by the FDA [3]. However, the levels of serum testosterone are lower in fifty percent of hip fractures occurred in elderly men. Endogenous testosterone and their metabolites play a role in maintaining bone health [4]. Regarding the issue of testosterone and/or the adrenal androgen dehydroepiandrosterone (DHEA), treatment of elderly men for preservation of bone and muscle mass remains an active area of debate and discussion. Based on the current state of uncertainty regarding testosterone treatment of aging men, the Institute of Medicine has recommended that a series of clinical trials be done to help determine the efficacy of testosterone for several important outcomes, including bone [5]. Oral testosterone undecanoate (TU) is the only oral form of testosterone replacement therapy and is available in many countries. Due to the most common adverse drug reaction (ADR) of testosterone supplementation for aging males is increase in serum prostate specific antigen (PSA), with a potential threat for developing prostate grand tumour cancer [6, 7], it is essential to find out a effective and safe testosterone supplementation protocol. However, there is so little study reports on evaluating the effect and safety of low-dose testosterone treatment for elderly male osteoporosis up till now. So we perform this study to evaluate the effects of low-dose TU treatment on bone mineral density (BMD) and biochemical markers of bone turnover in elderly male osteoporosis with low serum testosterone and to observe the side effects of low-dose TU on PSA and prostate grand.

## 2. Material and Methods

### 2.1. Inclusion and Exclusion Criteria of Participants

With reference to the World Health Organization (WHO) definition [8], subjects with a BMD of 2.5 SD lower than the peak mean of the same gender (T ⩽ −2.5) were determined as osteoporotic. Men (aged >60 years) were screened if they had a T-score less than or equal to −2.5 (if no prevalent vertebral fracture) or less than or equal to −2.0 (if one prevalent vertebral fracture) at the femoral neck, total hip, trochanter, or lumbar spine and more than −4.0 at all sites. We enrolled 186 elderly male osteoporosis (average age 68.2 ± 5.2 years) with low serum testosterone (serum T < 300 ng/dL).

The exclusion criteria were clinical or laboratory evidence of systemic disease; presence or history of vertebral, hip, or wrist fractures; other metabolic bone diseases; prostate grand tumour; cancer; poorly controlled diabetes with HbA1c ⩾10%; uncontrolled hypertension with blood pressure ⩾180/100 mmHg; uncontrolled hypothyroidism; uncontrolled hyperthyroidism; hyperparathyroidism; abnormal liver function with alanine aminotransferase (ALT) and aspartate aminotransferase (AST) values >2-fold upper limits, or renal disease with serum creatinine >2 mg/dL; the use of HT, selective estrogen (or androgen) receptor modulators, or the use of calcitonin, chronic systemic corticosteroid, or any other treatment affecting BMD within the previous 6 months; or any use of bisphosphonate within the previous 12 months. 

### 2.2. Study Design

This study was designed as a 2-year, single-center, randomized, placebo-controlled, open-label clinical trial. Patients were recruited from Changsha city and its surrounding area in Hunan province of China. All the participants were randomly divided into three group: standard-dose TU group (group A), low-dose TU group (group B), and placebo group (group C). The study protocol was approved by the second Xiangya hospital of central south university ethics committees in accordance with the Declaration of Helsinki and Good Clinical Practices Guidelines. 

### 2.3. Treatment Assignments

Each subject in the standard-dose TU group (group A, *n* = 62) took Andriol Testocaps when having breakfast (Testosterone Undecanoate, Merck Sharp and Dohmo Ltd., China) 40 mg per day. Each subject in the low-dose TU group (group B, *n* = 62) took Andriol Testocaps 20 mg per day (each 40 mg capsule was divided into two average capsules). The remaining subjects (group C, *n* = 62) took one placebo capsules every day. All patients were also supplemented with calcium (600 mg) and vitamin D3 (125 IU) daily. Follow-up period lasted 24 months. Participants were requested to maintain their habitual diet and exercise patterns.

### 2.4. Assessment Methods

#### 2.4.1. Areal Bone Mineral Density Assessment

The parameters including the projected areal bone mineral density (a BMD, g/cm^2^) were measured by DXA using QDR 4500A fan beam bone densitometer (Hologic Inc., Bedford, MA, USA), according to the manufacturer's recommended standard analysis procedures for the PA lumbar spine (vertebrae L2–L4) and hip femoral neck. A long-term (exceeding 15 years) coefficient of variation (CV) for the BMC and BMD was not greater than 0.40%.

#### 2.4.2. Vertebral Fractures Assessment

Lumbar spine X-rays for vertebral fractures were given to participants at baseline and 6, 12, 18, 24 months after randomization. Genant's vertebral fractures assessment method [9] was used.

#### 2.4.3. Laboratory Tests

After an overnight fast, venous blood was sampled to determine plasma glucose (FPG), glycosylated hemoglobin A1c(HbA1c), total cholesterol (TC), high-density lipoprotein cholesterol (HDL-CH), low-density lipoprotein cholesterol (LDL-CH), triglyceride (TG), creatinine (Cr), ALT, and AST at baseline and at 6, 12, 18, and 24 months (Automatic Analyzer 7600-020, Hitachi.) Serum bone-specific alkaline phosphatase (BAP, Beckman Access Ostase, Fullerton, CA, USA; interassay coefficient of variation CV = 9% and intra-assay CV = 4%) and urine collected for routine urinalysis and N-telopeptide of type 1 collagen (NTx, Vitros Immunodiagnostic Products, Ortho-Clinical Diagnostics, Buckinghamshire, UK; interassay CV = 10% and intra-assay CV = 5%) were examined at baseline and at 6, 12, 18, and 24 months. Serum concentrations of total testosterone (TT), free testosterone (fT), and estradiol (E2) were analyzed at baseline and at 6, 12, 18, and 24 months by chemical luminescence method. 

#### 2.4.4. Safety Assessment and Adverse Events

In addition to the aforementioned laboratory tests, the safety of the participants was further monitored by B-ultrasonography for prostate grand, by chemical luminescence method for serum prostate specific antigen (PSA). Adverse events were classified according to body system. Participants were asked about their symptoms at the clinics every 3 months.

### 2.5. Statistical Analysis

Descriptive data are given as the mean ± standard deviation (SD) for continuous variables. For continuous variables, differences in mean percentage changes from baseline between the two groups were evaluated by Student's *t*-test. A *P* value of 0.05 or less was considered statistically significant.

## 3. Results

No significant differences in age, body mass index (BMI), FPG, HbA1c, TC, TG, HDL-CH, LDL-CH, PSA, fPSA, lum-bar spine and femoral neck BMD, TT, fT, E2, BAP, uNTX/Cr, and volume of prostate grand by B-ultrasonography were found at baseline between the three groups (Table 1).

### 3.1. Percentage Changes in BMD and Vertebral Fractures throughout 24 Months of Treatment

**Figure 1:**
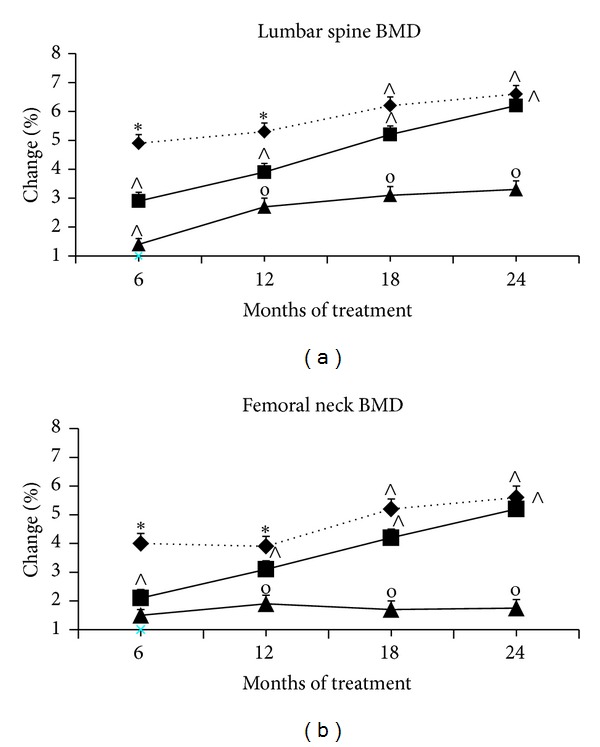
Changes in lumbar spine and femoral neck BMD throughout 24 months of treatment. Note: data are means ± SD. BMD: bone mineral density; ∗: *P* < 0.05  versus baseline, groups B and C; *∧*: *P* < 0.05  versus baseline and group C. ♦: group A, the standard-dose TU group; ■: group B, the low-dose TU group; ▲: group C, placebo group.

At baseline, lumbar spine and femoral neck BMD was similar in the three groups (Table 1). Since the 6th month followup in group A or since the 12th month followup in group B and throughout the study, lumbar spine and femoral neck BMD significantly increased (*P* < 0.05) compared with baseline and group C (Figure 1). In addition, at the 12th month followup, percentage of changes of lumbar spine and femoral neck BMD was significantly (*P* < 0.05) higher in group A than in groups B and C (Figure 1). Since the 18th month followup and throughout the study, the percentage of changes of lumbar spine and femoral neck BMD was significantly higher (*P* < 0.05) in groups A and B than in group C (Figure 1), and no significant differences were found between groups A and B since that time (Figure 1). None of the patients had vertebral fractures during two years' TU treatment.

### 3.2. Bone Metabolism Markers

**Figure 2:**
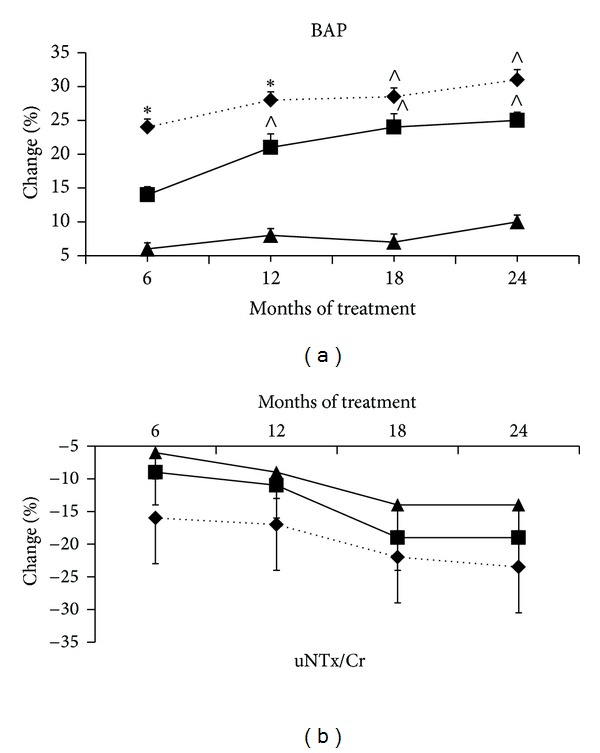
Changes in serum levels of BAP and uNTx/Cr throughout 24 months of treatment. Note: data are means ± SD. BAP: bone-specific alkaline phosphatase; uNTx/Cr: urine N-telopeptide of type 1 collagen/creatinine; ∗: *P* < 0.05  versus baseline, groups B and C; *∧*: *P* < 0.05  versus baseline and group C. ♦: group A, the standard-dose TU group; ■: group B, the low-dose TU group; ▲: group C, placebo group.

At baseline, the biochemical parameters of bone turnover were similar in the three groups (Table 1). Serum BAP levels significantly (*P* < 0.05) increased in group A since the 6th month followup and throughout the study and in group B since the 12th month followup and throughout the study (Figure 2). After 18 months of treatment, the percentage of increase in BAP levels was higher (*P* < 0.05) in groups A and B, without any difference between them, than in group C (Figure 2). Levels of uNTx/Cr were unchanged in all groups throughout the study period (Figure 2).

### 3.3. Sex Hormones

**Figure 3:**
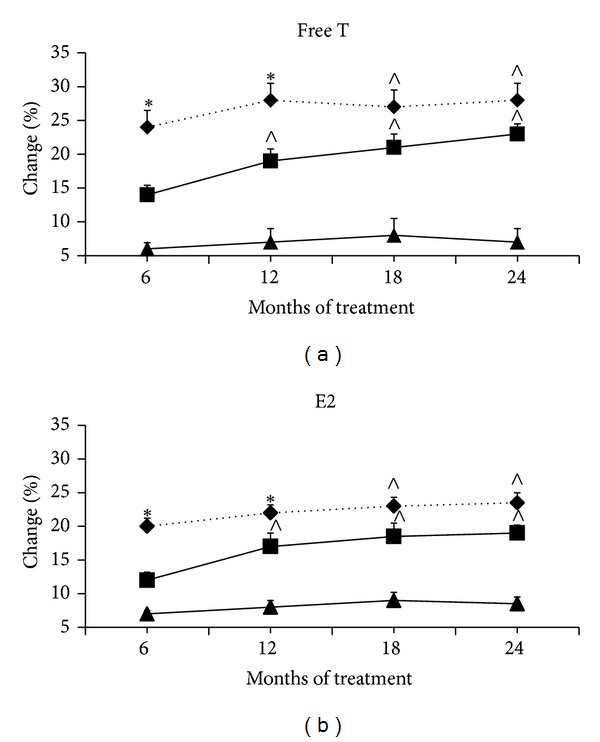
Changes in serum levels of free T and E2 throughout 24 months of treatment. Note: data are means ± SD. T: testosterone; E2: estradiol; ∗: *P* < 0.05  versus baseline, groups B and C; *∧*: *P* < 0.05  versus baseline and group C. ♦: group A, the standard-dose TU group; ■: group B, the low-dose TU group; ▲: group C, placebo group.

No significant differences in free T and E2 were found at baseline in all groups (Table 1). Serum-free T and E2 levels significantly (*P* < 0.05) increased in group A since the 6th month followup and throughout the study and in group B since the 12th month followup and throughout the study (Figure 3). After 18 months of treatment, the percentage of increase in free T and E2 levels was higher (*P* < 0.05) in groups A and B, without any difference between them, than in group C (Figure 3).

### 3.4. Side Effects and Dropouts

Throughout the 2-years observation, both doses of TU were equally well tolerated, and their safety profile was similar to that of placebo. There were no reports of nausea, vomiting, or diarrhea among study patients. All patients were monitored for serum levels of PSA, fPSA, and prostate grand by B-ultrasonography. There were no differences in these observed variables. No side effects on prostate glands were observed. There was also no significant difference in the incidence of all laboratory abnormalities between the three groups.

The numbers of withdrawals were similar in the three groups (five, four, and four men in groups A, B, and C). Dropouts were due to lack of compliance to the treatment in these patients. 

## 4. Discussion

Although there have been considerable advances in our understanding and management options for male osteoporosis, there are a number of important gaps in knowledge [10]. The results of the present study show for the first time the effects of low-dose TU on BMD in aged male osteoporosis with low serum testosterone. Our results show that after the 24-month treatment, lumbar spine and femoral neck BMD and serum BAP levels increased significantly in both of low-dose TU (20 mg, per day) or standard-dose TU (40 mg, per day) treatment groups. There were no significant differences between groups of low-dose TU and standard-dose TU in the percentage of changes of lumbar spine and femoral neck BMD and serum levels of free testosterone, estradiol, and BAP since the 18th month followup and throughout the study. However, there were no differences for levels of uNTX in all groups. The elderly male patients tolerated the two doses of TU quite well. Adverse effects were similar and slight in the two groups. No side effects on prostate glands including PSA were found. None of the patients had vertebral fractures during the two years' TU treatment.

In a recent study, Nair and colleagues [11] performed a 2-year placebo-controlled, randomized, double-blind study involving 87 elderly men with low levels of DHEA sulfate and bioavailable testosterone (defined as below the 15th percentile for young normal men). Over 2-year of treatment, men who received testosterone transdermally or DHEA had a modest (~2%) increase in BMD at the femur neck but not at the spine, total hip, or radius. Neither treatments had major adverse effects, including prostate-specific antigen levels. These findings using transdermal testosterone at doses that had only modest effects on serum testosterone levels contrast with previous studies that used im testosterone administration and achieved higher circulating testosterone levels [12]. Latter, testosterone therapy was associated with more clinically significant increases in bone mass. 

The age-related decline in testosterone level was attributed to two factors, which were the degeneration of Leydig's cells and the increase of SHBG level with age [13]. In vitro studies demonstrated that androgen could increase the proliferation and decrease the apoptosis of osteoblast via regulation of protein kinase B [14]. It also played a vital role in the process of mineralization, which is the late differentiation stage of osteoblast [15, 16]. Androgen also prevented parathyroid-induced osteoclast formation [17] and decreased bone resorption activity of osteoclast via deactivation of lysosomal enzymes [18].

 On the other hand, the traditional notion that estrogen is only important in maintenance of the female skeletal system while testosterone is vital for the male skeletal system is now challenged by several experiments of nature. Estrogen was found to be associated significantly with bone health status of elderly men in several large epidemiological studies. The Framingham study discovered that aged men with higher estradiol level had higher BMD, and the difference in BMD between the first quartile and the fourth quartile was equivalent to 10 years of aging on bone [19]. Positive and significant relationships between estradiol level and several hip strength parameters, especially cross-sectional area of bone, were also observed in the Boston Bone Health Study [20]. The estrogen hormone in men is produced via conversion of testosterone to estrogen via the aromatase enzyme (cytochrome 19) [21]. About 15% of the estrogen in men originates from the testes while the other 85% comes from peripheral tissue inclusive of bone. Furthermore, aromatase enzyme was found in osteoblasts, osteocytes, chondrocytes, and adipocytes but not in osteoclasts [22]. Therefore, it is reasonable to postulate that estrogen produced in the bone of men has paracrine or intracrine function [23].

 However, there are reports of metastatic prostate cancer after testosterone administration in (elderly) men [24, 25]. This has raised concern that testosterone-replacement therapy should be given to aging men who do not have significantly high risk of developing prostate cancer. The current Endocrine Society Guidelines have been developed to render testosterone administration to elderly men acceptably safe therapy in men without a prior history of prostate carcinoma or without evidence of harboring a prostate carcinoma [26]. The members of the working group agreed that because the normative ranges for TT and FT in healthy young men vary among laboratories and assays (lower TT limits: 280–300 ng/dL; lower fT limits: 5–9 pg/mL) [27], clinicians should use the lower limit of normal range for healthy young men established in their laboratory. Members of the working group disagreed on T concentrations below which testosterone supplementation should be offered to older men with symptomatic hypogonadism. Some members of the working group recommended T supplementation in older men with TT level below 300 ng/dL, symptoms that might be attributable to low testosterone; others recommended T supplementation only in those with TT level below 200 ng/dL, because higher pretreatment T values are associated with lower beneficial effects of T therapy. A study in a worldwide sample of 1,438 men has found that the most common adverse drug reaction of injectable TU for the treatment of male hypogonadism is an increase in serum PSA with a potential threat for developing prostate grand tumour cancer [7], so to develop an effective and safe testosterone supplementation protocol is essential.

## 5. Conclusion

Treatment with low-dose TU (20 mg, per day) in elderly male osteoporosis with low serum testosterone effectively increases lumbar spine and femoral neck BMD and improves their bone turnover, similar to treatment with standard-dose TU (40 mg, per day). No side effects on prostate glands including prostate specific antigen were found. Low-dose TU may be a cost-effective and safe protocol for treating elderly male osteoporosis. Further clinical trials of large-sample, multi-center and longterm on the efficacy and safety of low-dose testosterone undecanoate treatment in elderly male osteoporosis with low serum testosterone is need.

## Figures and Tables

**Table 1:** Characteristics of the patients at the entry (means ± SD).

Parameter	A	B	C
Regimen of treatment	TU 40 mg/d	TU 20 mg/d	Placebo 1 tablet/d
Patients (no)	62	62	62
Age (yr)	68.1 ± 5.4	68.4 ± 5.5	68.0 ± 4.8
Height (cm)	169.2 ± 2.7	168.3 ± 3.1	170.6 ± 2.5
Weight (kg)	82.0 ± 3.5	81.9 ± 4.8	84.1 ± 3.7
BMI (kg/m^2^)	27.9 ± 3.2	28.2 ± 3.6	28.7 ± 2.9
SBP (mmHg)	136.2 ± 15.8	138.5 ± 9.9	142.8 ± 12.8
DBP (mmHg)	82.1 ± 4.5	86.2 ± 5.6	87.1 ± 6.2
FPG (mmol/L)	6.7 ± 0.6	6.3 ± 0.5	6.5 ± 0.7
HbA1c (%)	6.6 ± 0.7	6.8 ± 0.6	6.4 ± 0.8
TC (mmol/L)	4.9 ± 1.2	4.4 ± 1.0	5.1 ± 1.6
TG (mmol/L)	2.9 ± 1.2	2.6 ± 1.3	3.1 ± 1.4
HDL-CH (mmol/L)	0.9 ± 0.2	0.9 ± 0.3	0.8 ± 0.2
LDL-CH (mmol/L)	3.3 ± 0.9	3.1 ± 0.6	3.4 ± 1.2
PSA (ng/mL)	3.9 ± 0.8	3.6 ± 1.0	3.7 ± 0.7
fPSA (ng/mL)	0.7 ± 0.2	0.6 ± 0.3	0.6 ± 0.2
BMD (g/cm^2^)			
Lumbar spine	0.783 ± 0.088	0.802 ± 0.085	0.797 ± 0.080
Femoral neck	0.598 ± 0.073	0.586 ± 0.076	0.592 ± 0.077
Sex hormones			
TT (ng/dL)	214.8 ± 22.4	218.3 ± 25.1	220.1 ± 20.7
fT (pg/mL)	4.2 ± 1.1	3.9 ± 0.9	3.8 ± 0.7
E2 (pg/mL)	10.8 ± 5.7	13.9 ± 7.6	15.3 ± 6.8
Bone turnover markers	33.6 ± 12.4	32.2 ± 10.9	30.9 ± 11.3
BAP (IU/L)	37.1 ± 3.4	36.0 ± 5.2	36.5 ± 5.1
uNTX/Cr (nmol/mmol)	6.9 ± 1.3	6.8 ± 1.0	6.6 ± 1.4

TU: testosterone undecanoate; BMI: body mass index; FPG: plasma glucose; HbA1c: glycosylated hemoglobin A1c; TC: total cholesterol; TG: triglyceride; HDL-CH: high-density lipoprotein cholesterol; LDL-CH: low-density lipoprotein cholesterol; PSA: prostate specific antigen; fPSA: free prostate specific antigen; BMD: bone mineral density; TT: total testosterone; fT: free testosterone; E2: estradiol; BAP: bone-specific alkaline phosphatase; uNTX/Cr: urine N-telopeptide of type 1 collagen/creatinine.
